# Effect of Marital Relationship Enrichment Program on Marital Satisfaction, Marital Intimacy, and Sexual Satisfaction of Infertile Couples

**DOI:** 10.22074/ijfs.2017.4885

**Published:** 2017-09-03

**Authors:** Seyedeh Zahra Masoumi, Somayeh Khani, Farideh Kazemi, Fatemeh Kalhori, Reyhaneh Ebrahimi, Ghodratollah Roshanaei

**Affiliations:** 1 Department of Midwifery, Mother and Child Care Research Center, Hamadan University of Medical Sciences, Hamadan, Iran; 2 Department of Midwifery, Student Research Center, School of Nursing and Midwifery, Hamadan University of Medical Sciences, Hamadan, Iran; 3 Department of Midwifery and Reproductive Health, School of Nursing and Midwifery, Shahid Beheshti University of Medical Sciences, Tehran, Iran; 4 Department of Biostatistics and Epidemiology, Modeling of Noncommunicable Diseases Research Center, School of Public Health, Hamadan University of Medical Sciences, Hamadan, Iran

**Keywords:** Infertility, Training, Marital Therapy, Sexual Satisfaction

## Abstract

**Background:**

Infertile couples only think of having children during their sexual intercourse, and their constant concern about this issue increases their stress level. Psychosocial and social stress leads to decreased life satisfaction, increased marital problems, and reduced sexual confidence. This study aims to determine the effect of enrichment program on marital and sexual satisfaction as well as marital intimacy among infertile couples.

**Materials and Methods:**

This randomized controlled clinical trial was conducted on 50 infertile couples in 2013 in Hamedan. The marital relationship enrichment program was taught to the experimental group during seven 90 minutes sessions. Enrich marital satisfaction, Linda Berg sexual satisfaction, and marital intimacy questionnaires were completed by both groups in 3 pretest steps immediately after the end of training sessions, and 8 weeks later. The results were analyzed in STATA11 software using t test, Chi-square, ANCOVA, RM-ANOVA, and Bonferroni post-hoc test. To check the data normality, Kolmogorov-Smirnov test was used. P<0.05 was considered significant.

**Results:**

Comparison of mean scores related to pretest on the one hand and immediately after the test in 8 week later on the other hand showed marital relationship enrichment program significantly increased marital and sexual satisfaction (P<0.001). Also, mean score of marital intimacy immediately after the test (P=0.04) and 8 weeks after the test (P<0.001) significantly increased in comparison with the pretest under the influence of the program.

**Conclusion:**

Enrichment training can increase marital intimacy and also marital and sexual satisfaction in infertile couples (Registration Number: IRCT201604299014N97).

## Introduction

Infertility fails to conceive after one year of sexual intercourse without using contraceptives methods (1). According to the World Health Organization’s report, infertility affects approximately 8 million couples around the world and its rate varies from 5 to 30% in different countries (2). According to Systematic Review and Meta-Analysis conducted on the infertility in Iran, the prevalence of primary infertility has been reported to be 10.6% (3). Infertility is considered as a sign of failure and implies that the person is not perfect. Most people do not think that they are infertile, so an infertility diagnosis is a shock for them (4). Anxiety, loss of self-esteem, shame, and depression caused by infertility damage the infertile couples' sexual function. Also, diagnosis, investigation, and treatment of infertility may interfere with their sexual satisfaction (5). It is estimated that 80% of marital conflicts and incompatibilities are caused by couples' lack of sexual satisfaction (6).

According to the results of Iranian reports, many couples suffer from lack of satisfaction in their sexual relations and 50 to 60% of divorces as well as 40% of sexual infidelity are caused by this factor (7). Infertility can be adversely associated with relational, sexual and psychosocial wellbeing (8). It has been reported that infertile couples only think about having children during their intercourse; therefore, constant concern about the issue of facing another failure leads to their increased level of stress (9). Psychosocial and social stress leads to decreased life satisfaction, increased marital problems, and reduced sexual confidence among infertile couples (10). Based on Berg and Wilson (4) conclusions marital adjustment is reduced with increasing number of years of infertility and marital distress created. Marital distress is affected by intra couple coping method (11). Marital difficulties in infertile men and women cause the self-blame and detachment (12) and marital functioning is decreased in infertile couples with treatment process (13, 14).

For this reason, physical infertility treatments are not enough by themselves, and paying attention to the mental needs of infertile couples is an essential element in infertility treatment (15). Most therapists regard training couples in communication skills as the first step to improve the performance of couples, because communication problems are the most widespread complaints expressed by the couples who seeking (16). There are multiple approaches preventing marital difficulties or improving marital compatibility, one of which is the marital relationship promotion program known as "marital preparation and enrichment" that was first introduced by Olson and Olson (17). This program is one of the most successful ones whose efficacy has been reported in different works (16, 18). The Enrichment program is one that seeks to improve couples’ relationships and could determine the factors and conditions upon which marital satisfaction and compatibility can be predicted after marriage. This program includes 4 preventive characteristics; first, it identifies the factors required by marital success. Second, it assists couples needing help to achieve growth and health criteria. Third, it requires feedback and training for the progress of couples. Finally, it provides some practices to couples that could affect their conflict resolution and communication skills. This program has 6 objectives and contains some exercises to help couples achieve these objectives with the purpose of encouraging them to do planning and cooperate with each other to deal with major issues (17).

Considering that a desirable sexual relationship can increase the chance of fertility (19). Infertility itself can be an important factor in marital dissatisfaction (20), and marital satisfaction can have a mutual influence on sexual satisfaction (21). This study was conducted to determine the effect of an enrichment program on marital and sexual satisfaction as well as marital intimacy of infertile couples using Enrich marital satisfaction, Linda Berg sexual satisfaction, and marital intimacy questionnaires in order to specify the effect of this preventive program on the satisfaction rate of infertile couples in terms of the expressed variables.

## Materials and Methods

In this randomized controlled clinical trial participants were selected from the infertile couples referring to IVF Center of Fatemieh Hospital, Hamedan, for treatment in 2013. Using data from the study by Choobforoushzadeh et al. (22), considering the sample size at confidence interval (CI) 95%, statistical power of 0.90, and sample loss, finally, 50 couples were selected (25 couples in the intervention group and 25 couples in the control group).

**Fig.1:**
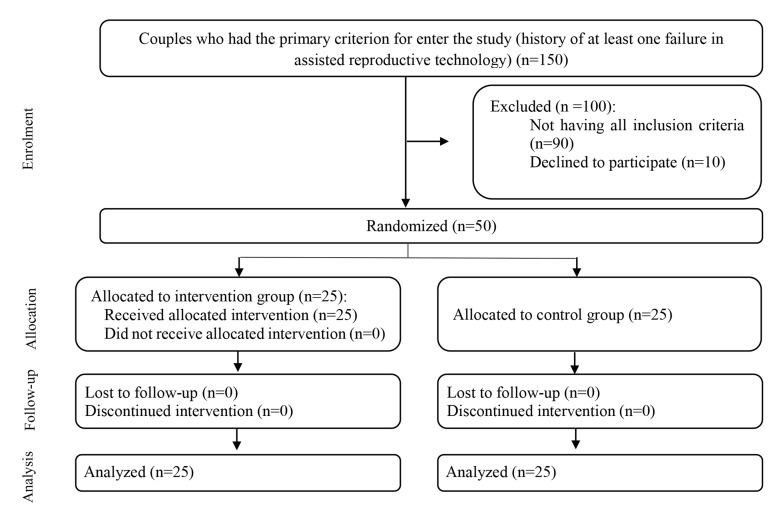
Flow diagram of the study.

First, the researcher prepared a list of the couples who had at least one history of failure in the use of assisted reproductive methods. After additional investigations, it was found that only 60 couples had all the inclusion criteria. From among these individuals, 50 couples who were willing to participate in the study were selected based on Helsinki principles (Fig .1). The random stratified sampling method was used to randomly assign these individuals into two experimental and control groups. For this purpose, the couples were first divided into two groups with monthly incomes of less than 5 million Rials and equal to or more than 5 million Rials. Then, they were divided into two subgroups in terms of infertility duration (less than 5 years and 5 years or more) and each of these subgroups was divided into three other subgroups according to education level (elementary, middle and high school, college). Finally, randomization was done based on drawing lots in the education sub-group and the participants were assigned into two studied groups with the ratio of 1:1. So, the assignment sequence was pre-determined. Assigning individuals to the groups was done by drawing lots and someone blind to the research, which led to proper concealment of assignment, but due to the intervention nature, blinding of the researcher and participants was not possible. Design, implementation, and reporting of the study were set based on the CONSORT statement (23).

Inclusion criteria included: i. Infertile couples with at least one failure history in infertility treatment using assisted reproductive methods, ii. Primary infertility, iii. Risk of infertility with female, male, both, and unknown factors, iv. Willingness to participate in the study, v. Having reading and writing literacy, and vi. Having less than 40 in marital satisfaction score in basis of Enrich marital satisfaction questionnaire. The participants were excluded from the study for the following reasons: lack of regular attendance at all sessions, attendance of only one of the marital partners, or pregnancy occurred during the treatment because the volunteers did not complete all of the survey instruments.

The data collection tool included demographic characteristics, Persian Enrich marital satisfaction, Linda Berg sexual satisfaction, and marital intimacy questionnaires. Persian Enrich marital satisfaction questionnaire contained 47 questions, 5 of which were related to children. Since the questionnaire was used for infertile men and women, these 5 questions were removed in the expert panel formed based on the related specialists (2 Ph.D. holders in reproductive health, 2 epidemiologists, 2 Ph.D. holders in health education, and 1 Ph.D. holder in nursing) and the infertile couples answered 42 questions. The validity of this questionnaire is confirmed in previous studies conducted in this field (8). The Linda Berg sexual satisfaction questionnaire has 17 questions, whose validity was confirmed by Salehi Fedardi (24). The marital intimacy questionnaire’s content validity was confirmed the Oulia et al. (25) study. Regarding reliability of each of the employed questionnaires, we obtained acceptable reliability for all tools examined by the Cronbach’s alpha coefficient analysis (Persian Enrich marital satisfaction, 0.87; Linda Berg sexual satisfaction, 0.91; and marital intimacy questionnaire, 0.85).

The participants in the study were contacted and asked to return to the center in order to complete the pretest questionnaires after assigning the individuals into two above-mentioned groups. The questionnaires were given to 100 people (50 men and 50 women) as intervention (25 men and 25 women) and control (25 men and 25 women) groups. The provisional norm of these questionnaires were calculated separately for the interventional and control groups. Before performing the research, written informed consent was obtained from all participants. One session of the expert panel was held, in which the respective professors attended (1 social prevention specialist, 1 statistician and epidemiologist, 1 psychiatrist, and 1 reproductive health specialist) to determine the best intervention method to promote the marital relationship of infertile couples. Finally, it was decided to use the couples' relationship enrichment model. For the experimental group and based on the couples' relationship enrichment model, training classes seven 90 minutes sessions as a group with couples (men and women at the same time) were held twice per week, which included indeed, the fourth session (training on sexual relationship promotion) was held separately for men and women (Table 1).

**Table 1:** Marital relationship enrichment program


First session	
Objective: Familiarity with the members and expression of the logic and objectives of the training sessions	
Educational content	
	Acquaintance with the participants
	Expression of goals
	Conclusion of a contract and getting a commitment for regular participation
Second session	
Objective: Open cognitive interpretation training	
Educational content	
	Studying the problem from the viewpoint of each infertile couple
	Making couples informed about kinds of irrational beliefs on infertility
	Training A-B-C principles in infertility Methods to deal with irrational beliefs on infertility
Third session	
Objective: Training intimacy between couples	
Educational content	
	Defining intimacy and its dimensions
	Training how to establish intimacy
	Practicing intimacy methods
	Feedback on the implementation of solutions
Fourth session	
Objective: Training on the improvement of sexual relationship	
Educational content	
	Expressing importance of sexual relationship
	Expressing cycle of sexual issues
	Factors hindering proper sexual relationship
	Diagnosis and intervention
	Training about wrong sexual myths
Fifth session	
Objective: Evaluating conflict resolution methods	
Educational content	
	Conceptual definition of marital conflict in infertility
	Understanding the normality of conflict between couples
	Extracting common ways of dealing with conflict among participants
	Training correct principles and practices of conflict resolution on infertility
	Practicing proper way of conflict resolution on infertility
Sixth session	
Objective: Conflict resolution via teaching problem solving	
Educational content	
	Effect of having self-attitude on the manner of infertility problem solving
	Identifying infertility problem solving process
	Steps of problem solving process
	Hindering factors of problem solving
Seventh session	
Objective: Home management training	
Educational content	
	Training how to deal with infertility problem
	Training how to deal with main families
	Training how to deal with financial problems of infertility
	Training how to deal with gender roles


In other words, six training sessions were held for men and women together, and the fourth session was held separately. The educational program was done by the researcher with Ph.D. degree in reproductive health along with a psychologist. Immediately after the end of the training sessions and 8 weeks later, sexual and marital satisfaction as well as marital intimacy questionnaires were given by someone who was not aware of the content of the training sessions and completed by both groups. A pamphlet containing the instructional materials of enrichment relations was presented to the control group after the follow-up completion in order to comply with the ethical issue.

### Statistical analysis

Results were analyzed in STATA 11 software using t test, Chi-square, ANCOVA, Repeated- Measure ANOVA, and Bonferroni post-hoc test. To check data normality, Kolmogorov-Smirnov test was used. P<0.05 was considered significant.

### Ethical considerations

This study was approved by the Medical Ethics Committee of Hamedan University and all participants gave an informed consent before commencing the study (code: IR.UMSHA.REC.1395.10).

## Results

Participant recruitment and follow-up began in September and ended in December 2013. Fifty patients (25 couples) participated in each one of the group. None of the participants were excluded from the study during the training and follow-up periods. Characteristics of the participants are compared in Table 2; no significant difference was found between the two groups.

**Table 2:** Baseline characteristics of participants by intervention and control groups


Variable		Intervention group	Control group	P value

Age (Y), mean ± SD		30.0 ± 4.9	28.3 ± 4.4	0.89^*^
Gender, n (%)				
	Male	25 (50)	25 (50)	1.00^**^
	Female	25 (50)	25 (50)	
Education level, n (%)				
	Primary	5 (10)	4 (8)	0.37^**^
	Secondary	6 (12)	12 (24)	
	High school and diploma	23 (46)	17 (34)	
	College	16 (32)	17 (34)	
Employment status, n (%)				
	Employed	27 (54)	25 (50)	0.63^**^
	Unemployed	23 (46)	25 (50)	
Residence, n (%)				
	City	40 (80)	40 (80)	0.34^**^
	Village	10 (20)	10 (20)	
Duration of marriage (Y), mean ± SD		6.7 ± 4.1	5.5 ± 2.1	0.08^*^
Duration of infertility (Y), mean ± SD		4.5 ± 3.9	4.1 ± 2.4	0.58^*^
The number of previous IVF, n (%)				
	1	43 (86)	40 (80)	0.13^**^
	2	5 (10)	2 (4)	
	3	2 (4)	4 (8)	
	4	-	4 (8)	
Cause of infertility, n (%)				
	Female	17 (34)	10 (20)	0.53^**^
	Male	17 (34)	16 (32)	
	Female-male	17 (34)	24 (48)	
Monthly income (1 million Rial), mean ± SD		6.8 ± 3.7	6.4 ± 1.7	0.42^*^


*; Independent t test, **; Chi-square test, and IVF; *In vitro* fertilization.

In this study, the P value for the Mauchly’s test of sphericity was not significant (P=0.62). So repeated measure test was used to compare the mean scores of marital and sexual satisfaction as well as marital intimacy at different times of investigation in both groups. Findings showed that the mean score of marital satisfaction had a significant difference (P<0.001) at different times between the two groups. The Bonferroni post-hoc test demonstrated statistically significant difference in marital satisfaction scores between immediately after completing the training courses and the pretest (P<0.001) as well as 8 weeks after completion of the training courses and the pretest (P<0.001). These results were repeated in the case of sexual satisfaction. Investigating the marital intimacy mean scores showed statistically significant difference between the experimental and control groups (P<0.001). The Bonferroni post-hoc test demonstrated that, immediately after the completion of the courses, the marital satisfaction score was significantly increased compared with the pretest (P=0.04). Also, the investigation conducted 8 weeks after the course completion showed significant increase of marital intimacy scores in comparison with the pretest (P<0.001, Table 3).

ANCOVA was used to eliminate the effect of pretest on the results obtained in the posttest. The findings showed that, by controlling for the pretest effect, the intervention significantly increased the marital and sexual satisfaction immediately after the intervention and 8 weeks later (P<0.001, Table 4).

**Table 3:** Comparing the intervention and control groups in terms of mean scores of marital satisfaction, sexual satisfaction and marital intimacy


		Before intervention(mean ± SD)	Immediately after intervention(mean ± SD)	2 months after intervention(mean ± SD)	P value^*^

Marital satisfaction					
	Control group	48.1 ± 8.4	43.4 ± 10.6	40.6 ± 10.3	<0.001
	Intervention group	42.9 ± 9.3	71.0 ± 1.0	62.6 ± 0.8	
	P value^**^	<0.001	<0.001	<0.001	
Sexual satisfaction					
	Control group	26.4 ± 11.0	39.3 ± 18.0	40.0 ± 19.4	<0.001
	Intervention group	57.8 ± 18.0	84.0 ± 1.2	83.4 ± 1.6	
	P value^**^	<0.001	<0.001	<0.001	
Marital intimacy					
	Control group	226.4 ± 43.2	224.9 ± 43.7	223.9 ± 44.3	<0.001
	Intervention group	301.4 ± 76.0	318.0 ± 77.2	423.2 ± 2.0	
	P value^**^	<0.001	<0.001	<0.001	


*; Repeated-Measure ANOVA and **; Independent t test.

**Table 4:** Results of ANCOVA investigating the relationship between grouping on marital satisfaction and sexual satisfaction


Measuring tool		Statistical indicators of variables	P value

Immediately after intervention			
	Marital satisfaction	Pretest	0.06
		Grouping	<0.001
	Sexual satisfaction	Pretest	<0.001
		Grouping	<0.001
Two months after intervention			
	Marital satisfaction	Pretest	0.01
		Grouping	<0.001
	Sexual satisfaction	Pretest	0.005
		Grouping	<0.001


## Discussion

The present study was performed to determine the effect of enrichment program on marital as well as sexual satisfaction and marital intimacy of infertile couples. As far as the effect of the program on marital satisfaction was concerned, individuals in the control group attained a higher mean score of marital satisfaction before the intervention, but after the test and two months later, these scores were decreased, which was probably due to the marital satisfaction effect of time and continued duration of infertility. The mean score in the individuals of the experimental group was higher immediately after the intervention in comparison with the pretest. Two months after the intervention, although this mean score was higher than the pretest, it was decreased compared with the study conducted immediately after the intervention.

A drop after 2 months proved the necessity of continuing the enrichment trainings. Providing continuous training either in person, through mass communication means, or by family, and friends, and other relatives could have a major role in increasing marital satisfaction. Maintenance and skills help couples maintain and apply what they have learned during the sessions and expand them to other areas of their life such as work environments (26). Laub et al. (27) concluded that the longer-term the enrichment program and the more emphasis on the formation of skills, the higher and more stable its positive effect on the couples and their life satisfaction would be. As mentioned, among infertile couples undergoing infertility treatment, couples relationship enrichment program could increase the level of marital satisfaction in the experimental group. The findings of this study were consistent with the results of other works (16, 26); in the study by Isanezhad et al. (26) conducting on 36 couples in Comprehensive Medical and Counseling Center in Isfahan, the relationship enrichment program could significantly improve the total score of couples’ marital quality, including marital agreement, satisfaction, and marital cohesion. This effect persisted in the follow-up carried out 1 month later. Ghasemi Moghadam et al. (16) in their study which was conducted on married women in Tehran during 2010 to 2011 observed that marital relationship enrichment training using Olson’s method could significantly increase the mean score of overall marital satisfaction, even when husbands did not participate in the training program.

The results of the current study showed that the marital relationship enrichment training was effective in increasing the infertile couples’ intimacy in the pretest and 2 months later. The finding that satisfaction with marital relationship could have an effect on marital intimacy was consistent with Etemadi’s finding in 2005 on the application of cognitive-behavioral techniques and increased marital intimacy (28). In another hypothesis by these authors representing the role of applying therapeutic communication techniques to increase marital intimacy, it was found that use of the therapeutic communication techniques could increase affective, psychological, intellectual, spiritual, social, and entertainment intimacy. This finding was consistent with our research results.

Another objective of the present study was to determine the effect of this program on the couples' sexual satisfaction. The marital relationship enrichment training increased sexual satisfaction in infertile couples. In support of this finding, Shams Mofarahe et al. (29) also demonstrated that the sexual satisfaction of women, 1 month after consultation, was higher in the intervention group than the control group. In fact, if sexual relations between spouses are not satisfactory, this leads to feelings of deprivation, frustration, insecurity and lack of happiness. Dissatisfaction with sexual relationship might cause problems such as depression (30) or divorce (31). Satisfactory sexual relations could contribute to family strengthening and become the basis for acquiring and consolidating a solid relationship (32). Laub et al. (27) investigated the effectiveness of a relationship enhancement program on couples’ relationships in the long run and concluded that the spouses attained higher sexual and physical intimacy as well as more communication stability than the control group. To prevent sample loss due to the large number of training sessions, the sessions were held twice per week.

## Conclusion

Considering the positive impact of the enrichment program at posttest and follow-up stages on marital and sexual satisfaction and sexual intimacy in the infertile couples, it can be concluded that the program can be appropriately used in infertile couples with sexual problems. Enrichment skills are the skills that help satisfy the strongest desires of families (sexual desires) and are used in almost all cultures. Considering the fact that enrichment training is a preventive and non-invasive program and can prevent deterioration into marital conflict, establishing and developing a center to provide such training is recommended, especially for vulnerable groups such as infertile couples who need special attention.
